# Association between muscle quality index and time to stand up from the prone position in community-dwelling older adults

**DOI:** 10.1371/journal.pone.0359858

**Published:** 2026-10-05

**Authors:** Álvaro Huerta Ojeda, Emilio Jofré-Saldía, Guillermo Barahona-Fuentes, Sergio Galdames Maliqueo, Maximiliano Bravo Yapur, Regina de Villa Garduño, Carlos Jorquera-Aguilera, María-Mercedes Yeomans-Cabrera, Estélio H. M. Dantas

**Affiliations:** 1 Núcleo de Investigación en Salud, Actividad Física y Deporte ISAFYD, Universidad de Las Américas, Viña del Mar, Chile; 2 Escuela de Ciencias de la Actividad Física, el Deporte y la Salud, Universidad de Santiago de Chile (USACH), Santiago, Chile; 3 Facultad de Educación y Ciencias Sociales, Universidad Andres Bello, Viña del Mar, Chile; 4 Laboratorio de Análisis de la Actividad Física y del Deporte, Facultad de Ciencias de la Actividad Física y del Deporte, Universidad de Playa Ancha de Ciencias de la Educación, Valparaíso, Chile; 5 Departamento Medicina Interna, Universidad de Valparaíso, Viña del Mar, Chile; 6 Facultad de Ciencias, Escuela de Nutrición y Dietética, Universidad Mayor, Santiago, Chile; 7 Facultad de Salud y Ciencias Sociales, Universidad de Las Américas, Providencia, Chile; 8 Programa de Pós-Graduação em Enfermagem e Biociências (PPgEnfBio), Universidade Federal do Estado do Rio de Janeiro (UNIRIO), Rio de Janeiro, Brazil; University Hospital of Padova, ITALY

## Abstract

Both muscle strength and muscle quality are essential factors for preserving autonomy and functionality in older adults (OA). However, the relationship between muscle quality index (MQI) and the time to stand up from the prone position (SPP) in this population is unknown. The primary purpose of this study was to determine the relationship between MQI and SPP completion time in community-dwelling OA capable of performing the test, considering age, sex, stature, and fat mass percentage. This cross-sectional study included 376 OA (70.47 ± 6.95 years; 234 women and 142 men). Functional Autonomy (FA) was assessed using the GDLAM protocol, and MQI was calculated as manual grip strength divided by body mass index (N/BMI). Pearson's test was used to correlate parametric data, while Spearman's was used for non-parametric data. For both the dominant (D) and non-dominant (N-D) limbs, the analysis showed a high correlation between MQI and time in the SPP test (D: *r* = −0.55, 95% CI −0.62 to −0.48, *p* < 0.0001; N-D: *r* = −0.57, 95% CI −0.64 to −0.50, *p* < 0.0001). This association remained significant in all four regression models after adjustment (all *p* < 0.001), with models based on body mass showing greater explanatory power than those based on body mass index (adjusted R² 0.351–0.366 vs. 0.343–0.360). These results indicate that higher MQI is associated with shorter SPP completion time in community-dwelling OA. Therefore, the SPP test shows potential as a practical, field-based indicator associated with FA in this population.

## Introduction

In recent decades, as in other regions of the world, the Chilean population has experienced an increase in life expectancy, with a mean of 80 years for women and 75 years for men [1]. This increase in years of life has specific implications for quality of life in advanced stages, especially for preserving independence [2,3]. Likewise, when comparing women with men of the same age, women tend to have poorer health status but longer life expectancy; this paradox of health and survival between women and men is likely related to a combination of biological, behavioral, and social factors [4]. In this context, several studies highlight the fundamental role of muscle strength and quality in preserving the autonomy and functionality of older adults (OA) [5,6]. Indeed, evidence shows that greater muscle strength is directly related to a greater ability to perform daily life activities (DLAs) without assistance [7]. Similarly, the Muscle Quality Index (MQI) is a particularly relevant indicator for evaluating the muscles' efficiency in DLAs [8]. This index is considered a key factor in OA since higher MQI values are associated with greater functional autonomy (FA) and more autonomous aging [9]. Therefore, higher MQI values could be associated with better overall well-being among older adults and with reduced risk of dependency in this population [8,10].

As mentioned, the MQI is directly related to the FA of OA. Specifically, FA refers to a person's ability to perform DLAs [11,12]. The loss of FA not only affects OA physically but also has a psychological impact, limiting participation in social activities and decreasing self-esteem, which contributes to the deterioration of life quality [13]. Evidence shows that the lack of FA is closely associated with increased demand for external care, implying emotional and economic costs for both OA and society [12]. Currently, different tests and batteries are used to evaluate FA in institutionalized OA [14] and community-dwelling OA [15–18]. Among these batteries is the protocol of the Latin American Development Group for the Maturity, known by the acronym GDLAM, derived from its original Portuguese name “*Grupo de Desenvolvimento Latino-Americano para a Maturidade*” [19]. This protocol comprises tests that assess the DLAs of OA, such as dressing, sitting, standing, and walking [16–18]. Specifically, one of the tests of the GDLAM protocol that involves strength, coordination, and postural control (fundamental abilities to face unexpected situations in daily life) is the “standing up from a prone position” (SPP) test, designed to assess the time to transition from a lying position to a standing position quickly.

In this context, the SPP test has demonstrated both validity [19] and reliability [16,18] for assessing a functional activity of daily living in OA, incorporating muscle strength, balance, coordination, and mobility during performance. Like the other tests in the GDLAM protocol, the SPP test is characterized by its ease of administration, low cost, rapid execution, and minimal equipment requirements. It also offers high clinical and community applicability, has no ceiling effect, and includes rating scales that facilitate the interpretation of functional performance in older adults [15,17,20]. Although previous research has highlighted the relationships among muscle strength, MQI, and FA [7,10], the relationship between MQI and functional tests, such as SPP, in community-dwelling OA has not yet been sufficiently explored. This knowledge gap is crucial, as understanding how the two variables are linked could guide preventive interventions more efficiently, promoting healthy aging. Consequently, the primary purpose of this study was to determine the relationship between MQI and SPP completion time in community-dwelling OA capable of performing the test, considering age, sex, stature, and fat mass percentage. Its specific objectives were (i) to compare SPP completion time between older women and men and (ii) to analyze the representativeness and explanatory power of different models that relate the MQI to the SPP test, taking age, sex, stature and fat mass percentage into account.

## Materials and methods

### Research design

The following study was developed in accordance with the Strengthening the Reporting of Observational Studies in Epidemiology (STROBE) [21,22]. The research had a comparative cross-sectional observational design [23]. The study employs a correlational design to analyze the relationship between the MQI and performance on the SPP test, using observable, quantifiable variables obtained through direct assessment in the outpatient setting. However, due to the study’s cross-sectional design, causal relationships cannot be established. The evaluations were carried out over three days, with a 24-hour interval between sessions. The first session included signing the informed consent form for all participants and evaluating cardiovascular parameters such as electrocardiogram (ECG) and blood pressure (BP), anthropometric evaluation, and manual grip strength. Finally, to safely include the participants, a geriatric assessment was performed. The second session was for familiarization with the five tests of the GDLAM protocol, and finally, during the third session, the application of the GDLAM protocol was performed (Fig 1).

### Participants

Three hundred seventy-six OA (234 older women aged 68.92 ± 6.84 years and 142 men aged 73.02 ± 6.35 years) voluntarily participated in the study. The inclusion criteria were as follows: a) being over 60.0 years of age, b) being self-sufficient without risk, and c) having the autonomy to give consent or, failing that, being represented by a family member or legal representative. The exclusion criteria were as follows: a) having a terminal disease, b) having an uncontrolled chronic disease, c) having pre-existing severe cardiovascular conditions or those detected by the study ECG evaluation, d) having severe pulmonary conditions, e) having fractures in the last three months, f) having neurodegenerative diseases, g) having cognitive impairment, and h) being physically unable to perform some of the proposed physical tests (Fig 1). Participant recruitment and data collection were conducted between April 1, 2024, and July 31, 2026. The Scientific-Ethical Committee of Universidad de Las Américas, Chile (registration number: CEC_PI_2023003) approved the study and informed consent. All procedures respected ethical standards consistent with the Declaration of Helsinki (updated in 2013) and the ethical standards for exercise and sports sciences [24].

**Fig 1:**
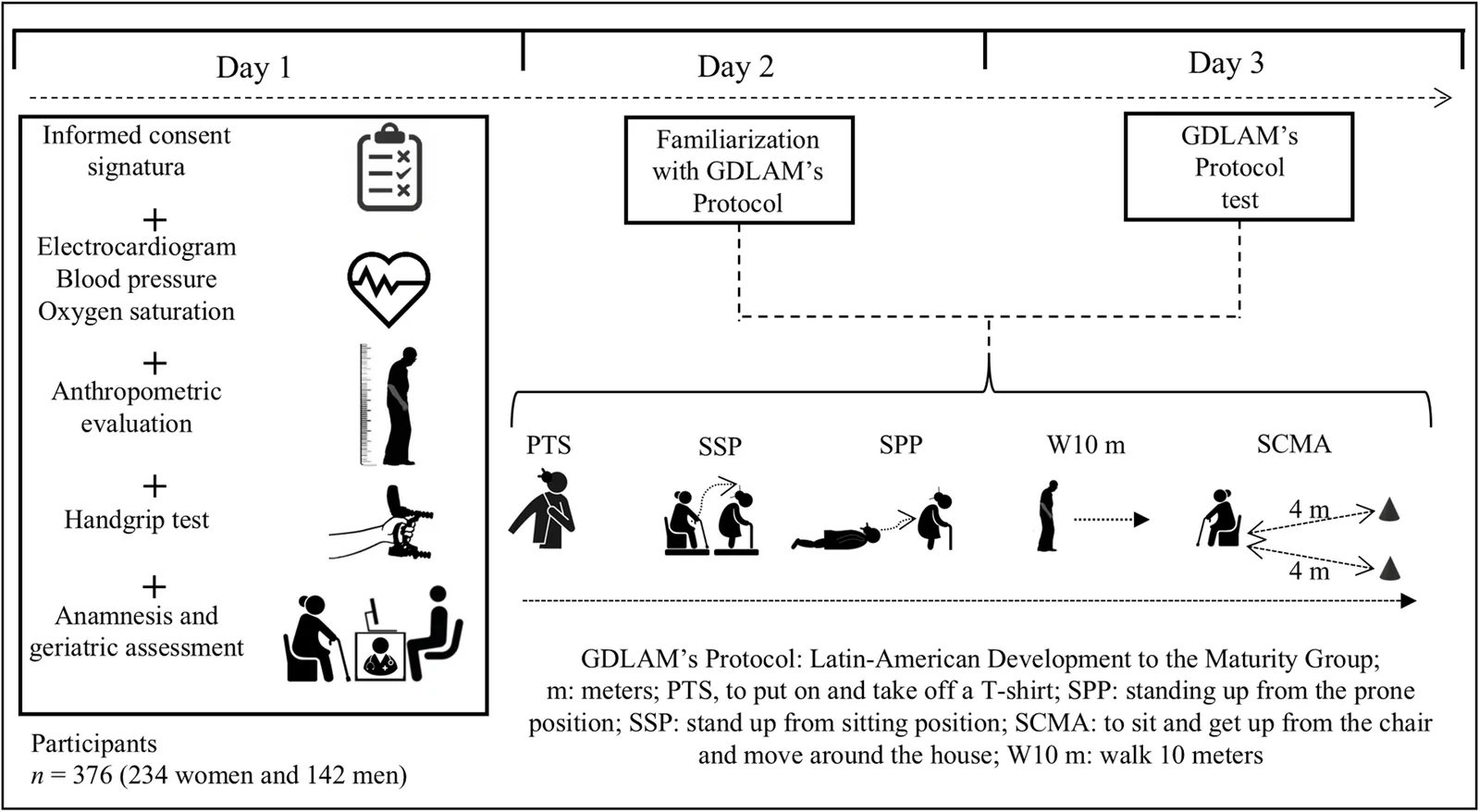
Research design.

### Electrocardiography and blood pressure

The variables obtained from the ECG and BP were not part of the study variables. However, they were determinant in safely including all participants in the study. BP was assessed in the left arm—OAs were placed in a sitting position, the left elbow was maintained at 125–135°, while the left forearm was supported on a table. BP was measured using a digital monitor (OMRON, model HEM-7120®, Kyoto, Japan). The resting ECG was performed in the lying, supine decubitus position. The ECG was performed with a 12-lead digital electrocardiograph with a wireless acquisition module (Mortara, model ELI 230®, Milwaukee, USA). The BP and ECG results, along with the geriatric evaluation, enabled the safe inclusion of OA in the subsequent stages of the study.

### Anthropometry: Body mass, stature, BMI, and fat mass

Body mass (BM) in kilograms (kg) was assessed using a digital scale (TANITA, model InnerScan BC-554®, Tokyo, Japan). Stature in meters (m) was measured using a wall-mounted measuring rod (SECA, model 700®, Hamburg, Germany). Both measurements were performed with the participants standing barefoot and their heads positioned in the Frankfurt plane, in accordance with the recommendations for basic anthropometric measurements [25]. Using these data, body mass index (BMI) was calculated as body mass (BM) divided by stature squared (kg/m^2^). The results were classified according to the World Health Organization's specific ranges for older adults [26]. The fat mass percentage (%) was determined using electrical bioimpedance, following the manufacturer's protocol (TANITA, model InnerScan BC-554®, Tokyo, Japan). This included individual settings for age, sex, and physical activity level. As the International Bioimpedance Protocol suggested, participants were instructed to avoid physical exercise, heavy meals, and fluid intake for 4 hours before measurement [27].

### Strength and MQI

A digital dynamometer (Jamar Plus + Hand dynamometer model; Sammons Preston Rolyan, Bolingbrook, IL, USA) was used to evaluate handgrip strength. The digital dynamometer was adjusted to the size of the participants’ hands [28]. Handgrip strength was assessed with the participants seated, with the arm at the side of the body, the elbow flexed at 90°, and the forearm in pronosupination. Maximal isometric handgrip was maintained for 3–5 seconds. Two measurements were performed for each hand: dominant (D) and non-dominant (N-D), with a 3-minute rest interval between tests on the same hand. The force generated was measured in Newtons (N). Then, for both D and N-D hands, the MQI was determined through two formulas: a) manual grip force divided by BMI (N/BMI), and b) manual grip force divided by BM (N/BM). Using two formulas to calculate the MQI enables analysis of handgrip strength adjusted for both BM and BMI, thereby standardizing muscle performance. This approach allows for more accurate identification of the model with the highest representativeness and explanatory power that links the MQI to the SPP test, while accounting for age and sex. Subsequently, for statistical analysis, the results of these two models—which included the D and N-D limbs—were correlated with the five tests and the autonomy index (GI) from the GDLAM protocol.

### GDLAM’s protocol

The Latin American Group for Maturity (GDLAM) protocol was used to assess the FA of OA [19]. This protocol considers the application of five functional tests (Fig 1). The five functional tests were described by Huerta Ojeda et al. [16] and Jofré-Saldía et al. [29]: a) To put on and take off a T-shirt (PTS), b) Stand up from the sitting position (SSP), c) Standing up from the prone position (SPP), d) Walk 10 meters (W10 m), and e) Sit and get up from the chair and move around the house (SCMA). The unit of measurement for these tests was timed in seconds (s). The rest interval between trials was five minutes. Then, the results of the five tests were used to calculate the GI using the following formula: GI = [(PTS + SSP + SPP + W10 m) × 2 + SCMA]/4.

The instruction manual for the full version of the GDLAM protocol can be accessed at: https://openrit.grupotiradentes.com/xmlui/bitstream/handle/set/7420/GDLAM_INGLE%cc%82S%20.pdf?sequence=2&isAllowed=y

### Data analysis

Data on age, anthropometry, FA, and muscle strength were recorded in an Excel spreadsheet designed for the study. The data are described using mean values and standard deviations (mean ± SD). The normality of the data was tested using the Kolmogorov-Smirnov test (*p* > 0.05). For parametric data, the comparison of variables between women and men was performed with a *t*-*t*est for independent samples. In contrast, a comparison of men and women was performed using the Mann-Whitney test for nonparametric data. After adjusting for the 16 between-group comparisons in Table 1 to control the false-positive risk (0.05 / 16 = 0.003) [30], the alpha level for these comparisons was set to *p* < 0.003. For the 24 correlations presented in Table 2, a separate Bonferroni correction was applied (0.05 / 24 = 0.002), so the alpha level for these correlations was set to p < 0.002. The Pearson test was used to correlate the parametric data, while Spearman’s test was used for the nonparametric data [31]. In both cases, 95% confidence intervals are presented. The criteria for interpreting the strength of the r coefficients were as follows: < 0.1 = trivial, 0.1–0.3 = small, 0.3–0.5 = moderate, 0.5–0.7 = high, 0.7–0.9 = very high, or > 0.9 = practically perfect [31]. Subsequently, a multiple linear regression analysis was conducted to examine the relationship between the MQI and the time to stand up from a prone position (SPP test); this analysis included the participants’ age, sex, stature, and fat mass percentage as covariates. Four models were considered in the analysis: a) N/BMI for the dominant hand, b) N/BMI for the non-dominant hand, c) N/BM for the dominant hand, and d) N/BM for the non-dominant hand. The criteria for interpreting linear regression (R²) were as follows: very low (< 0.02), low (0.02–0.13), moderate (0.13–0.26), or high (> 0.26) [32]. For each of the four regression models, independence of residuals was assessed with the Durbin-Watson statistic, multicollinearity with the variance inflation factor (VIF) and tolerance [33,34], normality of the standardized residuals with the Kolmogorov-Smirnov and Shapiro-Wilk tests, and homoscedasticity by visual inspection of the scatterplot of standardized residuals against standardized predicted values [34,35]. The significance level for these statistical analyses was *p* < 0.05. All statistical analyses were performed using SPSS software, version 21.0.0.0, for Windows®.

**Table 1:** Comparison of the age, anthropometry, GDLAM’s protocol, and MQI between women and men.

	All (*n* = 376)	Women (*n* = 234)	Men (*n* = 142)	Diff	95% CI of diff	*t /* U	*p*-value
Age and anthropometry							
Age (years)*	70.47 ± 6.95	68.92 ± 6.84	73.02 ± 6.35	4.43	3.05 to 5.78	10479	< 0.0001
Body mass (kg)*	72.93 ± 13.53	69.23 ± 12.56	79.04 ± 12.87	9.40	7.00 to 11.70	9050	< 0.0001
Stature (m)**	1.58 ± 0.08	1.52 ± 0.05	1.66 ± 0.06	0.13	0.12 to 0.15	22.48	< 0.0001
BMI (kg/m^2^)*	29.19 ± 4.73	29.65 ± 4.99	28.43 ± 4.17	−1.10	−2.00 to −0.20	14062	0.012
GDLAM’S protocol							
PTS (s)*	13.51 ± 4.22	12.71 ± 3.46	14.84 ± 4.96	1.77	1.01 to 2.52	11957	< 0.0001
SSP (s)*	9.84 ± 2.16	9.59 ± 1.82	10.25 ± 2.57	0.46	0.01 to 0.92	14554	0.043
SPP (s)*	4.58 ± 2.44	4.94 ± 2.53	3.98 ± 2.16	−0.72	−1.00 to −0.45	11185	< 0.0001
W10 m (s)*	6.19 ± 1.03	6.24 ± 1.00	6.11 ± 1.07	−0.14	−0.32 to 0.04	15057	0.127
SCMA (s)*	38.98 ± 7.37	39.24 ± 6.81	41.20 ± 8.09	1.59	0.26 to 2.87	14181	0.017
GI (points)*	27.06 ± 5.37	26.56 ± 4.95	27.89 ± 5.90	1.08	0.08 to 2.21	14434	0.032
Muscle quality index							
Strength D (N)**	269.3 ± 77.6	227.1 ± 46.1	338.8 ± 68.5	111.6	99.9 to 123.3	18.85	< 0.0001
Strength N-D (N)**	255.5 ± 75.6	216.5 ± 45.6	319.9 ± 71.3	103.4	91.5 to 115.2	17.13	< 0.0001
MQI D (S/BMI)**	9.46 ± 3.11	7.86 ± 2.03	12.10 ± 2.77	4.23	3.75 to 4.72	17.02	< 0.0001
MQI N-D (S/BMI)**	8.97 ± 3.04	7.47 ± 1.94	11.43 ± 2.91	3.94	3.45 to 4.44	15.74	< 0.0001
MQI D (S/BM)**	3.73 ± 0.98	3.36 ± 0.81	4.34 ± 0.90	0.98	0.80 to 1.16	10.85	< 0.0001
MQI N-D (S/BM)**	3.54 ± 0.96	3.19 ± 0.77	4.10 ± 0.96	0.90	0.72 to 1.08	9.96	< 0.0001

** For the comparison between groups, the t-test was applied. The difference between means and its 95% CI are reported, and t is the Student’s t-test statistic. * For the comparison between groups, the Mann-Whitney test was applied. The Hodges-Lehmann estimator of the between-group difference and its 95% CI are reported, and U is the Mann-Whitney test statistic. The alpha level for all statistical analyses was *p* < 0.003. BM: body mass; BMI: body mass index; CI: confidence intervals; cm: centimeters; D: dominant; diff: difference; GDLAM’S Protocol: Latin-American Development to the Maturity Group; GI: GDLAM index of autonomy; kg: kilograms; kg/m^2^: kilograms per square meter; m: meters; MQI: muscle quality index; N: Newtons; N-D: non-dominant; PTS: to put on and take off a T-shirt; s: seconds; S: strength in Newtons; SCMA: to sit and get up from the chair and move around the house; SPP: standing up from the prone position; SSP: stand up from sitting position; *t*: Student's t-test statistic; U: Mann-Whitney U test statistic; W10 m: walk 10 meters.

**Table 2:** Correlation between different MQI equations and GDLAM’S protocol.

All (*n* = 376)	PTS (s) *r* 95% CI	SSP (s) *r* 95% CI	SPP (s) *r* 95% CI	W10 M (s) *r* 95% CI	SCMA (s) *r* 95% CI	GI (points) *r* 95% CI
N/BMI D	−0.05 −0.15 to 0.05	−0.09 −0.19 to 0.01	−0.55^*^ −0.62 to −0.48	−0.32^*^ −0.41 to −0.22	−0.22^*^ −0.32 to −0.12	−0.24^*^ −0.34 to −0.14
N/BMI N-D	−0.10 −0.20 to 0.00	−0.11 −0.22 to −0.01	−0.57^*^ −0.64 to −0.50	−0.40^*^ −0.48 to −0.31	−0.28^*^ −0.37 to −0.18	−0.30^*^ −0.39 to −0.20
N/BM D	−0.13 −0.23 to −0.02	−0.15 −0.25 to −0.04	−0.58^*^ −0.64 to −0.51	−0.32^*^ −0.41 to −0.23	−0.29^*^ −0.38 to −0.19	−0.31^*^ −0.40 to −0.22
N/BM N-D	−0.19^*^ −0.29 to −0.09	−0.18^*^ −0.28 to −0.08	−0.61^*^ −0.67 to −0.54	−0.41^*^ −0.50 to −0.32	−0.35^*^ −0.44 to −0.26	−0.38^*^ −0.47 to −0.29

BM: body mass; BMI: body mass index; CI: confidence intervals; D: dominant; GDLAM’S Protocol: Latin-American Development to the Maturity Group; GI: GDLAM index of autonomy; MQI: muscle quality index; N: Newtons; N-D: non-dominant; PTS: to put on and take off a T-shirt; s: seconds; SCMA: to sit and get up from the chair and move around the house; SPP: standing up from the prone position; SSP: stand up from sitting position; W10 m: walk 10 meters; *: statistically significant after Bonferroni correction for the 24 correlations in this table (*p* < 0.002 = 0.05/24).

## Results

### Age, anthropometry, GDLAM protocol, and MQI

At the time of the study, the 376 participants had a mean age of 70.47 ± 6.95 years and a BMI of 29.19 ± 4.73 (kg/m^2^). Regarding the GDLAM protocol, specifically the PTS test, the mean time was 13.51 ± 4.22 s, with a significant difference between women and men (*p* < 0.0001). In the SPP tests, the mean performance time was 4.58 ± 2.44 seconds, with a significant difference between women and men (*p* < 0.0001). The participants showed higher MQI values, as measured by different metrics (N/BMI and N/BM), in the dominant limb than in the non-dominant limb. In addition, men had a significantly higher MQI than women across all evaluated metrics (*p* < 0.0001). For example, for N/BMI of the dominant limb, men had a mean of 12.10 ± 2.77, whereas women had 7.86 ± 2.03 (Table 1).

### Correlation between hand grip strength and GDLAM’s protocol

The analysis of the correlation between the hand grip strength of the dominant limb and the time in the SPP test revealed a moderate correlation (*r* = -0.48, CI 95%: -0.55 to -0.39, *p* < 0.0001). This moderate negative correlation was consistent among women (*r* = -0.47, 95% CI: -0.57 to -0.36, *p* < 0.0001), whereas a small correlation was observed among men (*r* = -0.27, 95% CI: -0.42 to -0.11, *p* = 0.0008). Similarly, the analysis of the correlation between non-dominant hand grip strength and time in the SPP test showed a strong correlation (*r* = -0.50, 95% CI: -0.57 to -0.41, *p* < 0.0001). When analyzing the group of older women, a correlation coefficient of *r* = -0.49 (95% CI: -0.58 to -0.38; *p* < 0.0001) was observed, whereas in the men, a correlation coefficient of *r* = -0.32 (95% CI: -0.47 to -0.16; *p* < 0.0001) was observed. The correlations between hand grip strength and time in the SPP test are presented in Fig 2 (panels A, B, C, D, E, and F).

**Fig 2:**
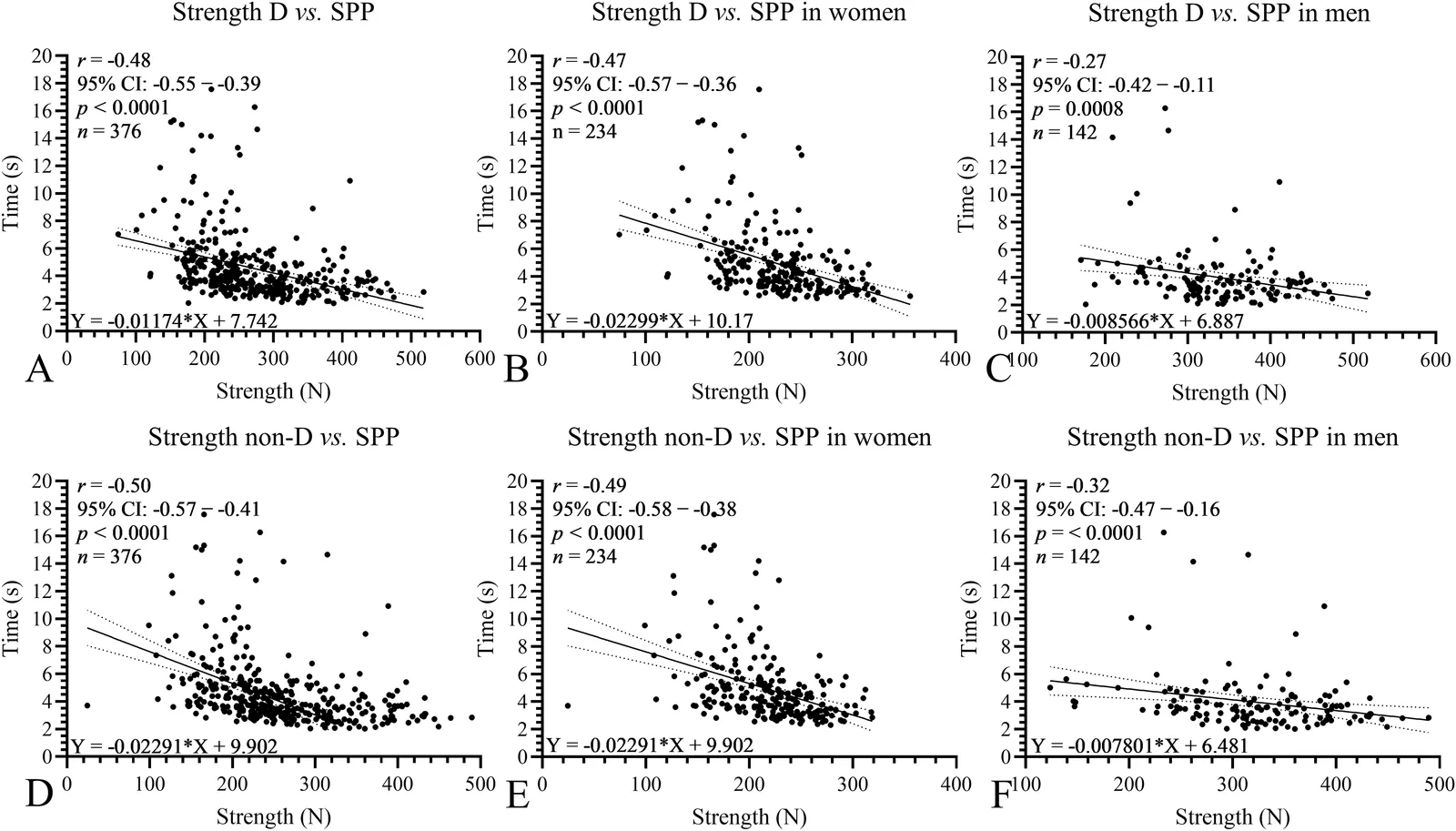
Correlation between hand grip strength and standing up from the prone position test. CI: confidence intervals; D: dominant; non-D: non-dominant; N: Newtons; p: p-value; s: seconds; SPP: standing up from the prone position.

### Different metrics for calculating MQI and their relationship with GDLAM’s protocol

When correlating the MQI (across all its metrics) with the GDLAM protocol tests, a negative correlation was observed with test times. The SPP tests stand out for their high correlation and statistical significance (*r* -0.55 to -0.61, *p* < 0.0001). The detailed analysis of the MQI values obtained using the different equations and their correlation with the GDLAM protocol is presented in Table 2.

### Multiple linear regression models examining the relationship between the MQI and the time to stand up from the floor, adjusted for age, sex, stature, and fat mass percentage

The assumptions of the four regression models were verified. Independence of residuals was adequate in all models (Durbin-Watson: 1.995–2.013), and no evidence of problematic multicollinearity was found (VIF: 1.21–3.16; tolerance: 0.317–0.827). However, the standardized residuals departed from normality in all four models (Kolmogorov-Smirnov and Shapiro-Wilk tests, both *p* < 0.001), showing positive skewness (2.31–2.42) and leptokurtosis (8.14–8.33). The scatterplots of standardized residuals against standardized predicted values also showed a funnel-shaped pattern suggestive of heteroscedasticity in all four models. These departures from normality and homoscedasticity are common in time-based physical performance outcomes, which are positively skewed, and are addressed as a limitation of the study (Table 3).

**Table 3:** Normality, skewness, and kurtosis of the standardized residuals of the multiple linear regression models.

Model	K-S	gl	Sig.	S-W	gl	Sig.	Skewness	Kurtosis
Model 1	0.145	376	< 0.001	0.807	376	< 0.001	2.317	8.142
Model 2	0.155	376	< 0.001	0.786	376	< 0.001	2.417	8.330
Model 3	0.142	376	< 0.001	0.809	376	< 0.001	2.308	8.180
Model 4	0.156	376	< 0.001	0.789	376	< 0.001	2.398	8.289

K-S: Kolmogorov-Smirnov statistic (Lilliefors significance correction); S-W: Shapiro-Wilk statistic; gl: degrees of freedom; Sig.: significance level. Skewness and kurtosis values correspond to the standardized residuals of each model.

Multiple linear regression models revealed significant associations between various muscle quality indices (MQI) and the time to stand up from the floor (SPP). All models were statistically significant and explained between 35.2% and 37.5% of the variance in SPP. In all four models, the MQI indices showed significant inverse associations with SPP: MQI N/BMI D (*β* ranging from -0.299 to -0.400, *p* < 0.001). Age was positively and significantly associated with SPP (*β* = 0.435-0.447, *p* < 0.001). Models 3 and 4 (MQI = N / BM) had greater explanatory power than Models 1 and 2 (MQI = N / BMI). Likewise, sex showed significant associations across all four models, though with smaller effect sizes than age and the MQI indices. Stature and fat mass percentage were also included as covariates; both were independently associated with SPP time in the models based on N/BMI, but not in the models based on N/BM. The regression analysis values are presented in Table 4.

## Discussion

The present study aimed to determine the relationship between MQI and SPP completion time in community-dwelling OA, considering age, sex, stature, and fat mass percentage, and to demonstrate that higher upper-extremity muscle quality (D and N-D) is associated with shorter time to stand up from the floor. The study also found that men performed better than women on the SPP test (*p* < 0.0001). However, no significant differences in GI were observed between the two sexes (*p* > 0.003). Finally, the two equations used to model the relationship between MQI (MQI = N / BMI and MQI = N / BM) and the SPP test, while controlling for age, sex, stature, and fat mass percentage, demonstrated high representativeness and explanatory power.

**Table 4:** Multiple regression models to determine the relationship between muscle quality indices, age, sex, stature, and fat mass percentage and the time to stand up from the prone position.

Model 1	R² R² corr.	F	*p*	MQI N/BMI D B 95% CI *β*, *p*	Age B 95% CI *β*, *p*	Sex B 95% CI *β*, *p*	Stature B 95% CI *β*, *p*	Fat mass B 95% CI *β*, *p*
SPP	0.369 0.360	43.236	< 0.001	−0.313 −0.415 to −0.212 −0.400, < 0.001	0.157 0.125 to 0.189 0.446, < 0.001	0.878 0.153 to 1.604 0.175, 0.018	0.046 0.008 to 0.083 0.167, 0.017	0.033 0.004 to 0.063 0.100, 0.028
Model 2	R² R² corr.	F	*p*	MQI N/BMI N-D B 95% CI *β*, *p*	Age B 95% CI *β*, *p*	Sex B 95% CI *β*, *p*	Stature B 95% CI *β*, *p*	Fat mass B 95% CI *β*, *p*
SPP	0.352 0.343	40.218	< 0.001	−0.268 −0.371 to −0.165 −0.334, < 0.001	0.157 0.124 to 0.190 0.447, < 0.001	1.049 0.321 to 1.777 0.209, 0.005	0.039 0.001 to 0.077 0.142, 0.043	0.039 0.009 to 0.069 0.117, 0.012
Model 3	R² R² corr.	F	*p*	MQI N/BM D B 95% CI *β*, *p*	Age B 95% CI *β*, *p*	Sex B 95% CI *β*, *p*	Stature B 95% CI *β*, *p*	Fat mass B 95% CI *β*, *p*
SPP	0.375 0.366	44.351	< 0.001	−0.856 −1.120 to −0.592 0.342, < 0.001	0.153 0.121 to 0.185 0.436, < 0.001	0.836 0.114 to 1.559 0.166, 0.023	0.009 −0.026 to 0.044 0.031, 0.628	0.029 −0.001 to 0.059 0.086, 0.061
Model 4	R² R² corr.	F	*p*	MQI N/BM N-D B 95% CI *β*, *p*	Age B 95% CI *β*, *p*	Sex B 95% CI *β*, *p*	Stature B 95% CI *β*, *p*	Fat mass B 95% CI *β*, *p*
SPP	0.359 0.351	41.522	< 0.001	−0.761 −1.030 to −0.491 −0.299, < 0.001	0.153 0.120 to 0.186 0.435, < 0.001	0.981 0.255 to 1.708 0.195, 0.008	0.008 −0.027 to 0.044 0.031, 0.641	0.034 0.003 to 0.064 0.102, 0.029

*β:* standardized coefficient; BM: body mass; BMI: body mass index; D: dominant; F: ANOVA test statistic for the model; MQI: muscle quality index; N: Newtons; N-D: non-dominant; *ns*: non-significant; *p:* significance level; R²: coefficient of determination; SPP: standing up from the prone position. Models are adjusted for age, sex, stature (m), and fat mass percentage.

### MQI and its association with FA

Our results showed that the MQI, calculated from manual grip strength and BMI, is strongly associated with the time to stand up from the floor. In this sense, higher muscle quality in the upper extremities (D and N-D) is associated with a shorter time to perform this task. The relationship between muscle quality and the time to stand up from the floor in older adults may be associated with the force generated by the upper limbs to perform this everyday task correctly, regardless of the technique used [36]. In general, evidence shows that muscle strength—represented in this study by the MQI—and physical performance are predictors of decline in ADLs [37]. In addition, the manual grip test is recognized for its association with function in OA [7]. Indeed, as evidenced in the present study, greater manual grip strength is positively associated with the SPP test; however, greater muscle strength and reduced asymmetry between segments may decrease the risk of falls with adverse outcomes [38]. Therefore, this relationship makes it a valuable predictor of overall functional capacity in OA. In parallel, some studies have linked muscle asymmetries to FA in older women, indicating that lower- and upper-extremity asymmetry is positively related to overall FA in older women living in the community [39]. As for the MQI, it has been shown that there is a strong relationship between the MQI and performance on the SPP test in OA (in this study, *r* = -0.51 to -0.61), even more so in OA with hip osteoarthritis (*r* = -0.76) [6]. Although during the present study, there was no intervention with physical exercise programs, it is essential to mention that the practice of physical activity, especially in a programmed, constant, and permanent way over time, is part of the non-pharmacological strategies that allow slowing physical deterioration, preventing sarcopenia and dynapenia, and maintaining functional autonomy in the population [3,40]. For example, it has been shown that including physical exercise programs increases functional capacity, physical capacity, quality of life, and motivation to exercise in OA living in the community [41]. The results of this study highlight the importance of incorporating tests such as SPP, which offer high reproducibility and agreement [42], are reliable and valid [16,18], include grading scales [15,17,20], and are simple and easy to apply. Likewise, it has been shown that incorporating strength-training programs significantly improves rehabilitation after highly invasive surgeries, such as total knee arthroplasty in OA [43]. In this sense, given its association with OA-related functional capacity, the results underscore the importance of maintaining a high MQI in this population, particularly in upper-extremity muscle quality. This may support the design of timely and efficient interventions aimed at mitigating functional deterioration and improving cognitive function and quality of life in this population [44].

In addition to age and sex, the expanded regression models showed that stature and fat mass percentage were independently associated with SPP time, but only in the models based on MQI = N/BMI (models 1 and 2), not in those based on MQI = N/BM (models 3 and 4). From a biomechanical perspective, greater stature implies a greater shift in the body’s center of mass during the transition from the prone to the standing position [45], which could partly explain the longer SPP times observed as stature increases. Similarly, a higher percentage of fat mass has been associated with poorer muscle quality and impaired functional performance in older adults, consistent with the concept of sarcopenic obesity [46,47], which could explain its association with SPP time. However, this interpretation should be viewed with caution, since the N/BMI formula inherently incorporates stature, given that BMI is calculated based on body mass and stature; therefore, part of the observed association between stature and SPP time in Models 1 and 2 may reflect this shared mathematical component rather than a purely independent biomechanical effect, which is consistent with the stronger correlation observed between stature and the N/BMI MQI compared to the N/BM MQI. Regardless of this consideration, the MQI remained the strongest and most consistent predictor of SPP duration across all four models, alongside age, reinforcing its validity as a functional indicator in this population, even after accounting for these additional anthropometric parameters.

### Sex differences and FA

While the pathophysiological pathways leading to frailty are not well defined, sex appears to be associated with the aging trajectory [4]. In this sense, it has been identified that the decrease in estrogen levels during menopause contributes to an accelerated loss of muscle mass and function in women [48], events that may be associated with MQI and, consequently, with performance in the SPP test. These differences could be associated with men's anatomy and reproductive function. For this reason, the cut-off points for their classifications are different [49]. Indeed, during the functional tests conducted in the present investigation, men performed better than women on the SPP and PTS tests (*p* < 0.0001). Likewise, men had greater manual grip strength than women, for both the dominant and non-dominant limbs (*p* < 0.0001); men also showed a greater MQI calculated using any of the equations (*p* < 0.0001). The shorter times performed by men in the functional tests, especially the SPP (women: 4.94 ± 2.53 s. men: 3.98 ± 2.16, *p* < 0.0001), the greater manual grip strength, and the greater MQI, suggest that these variables are associated with activities of daily living in OA and, consequently, should be addressed by individualized training programs to maintain satisfactory functioning of OA [50]. In this context, evidence suggests that women achieve greater reductions in cardiovascular mortality than men with lower levels of physical activity—achieving equivalent benefits with 140 minutes per week compared to 300 minutes for men—which suggests that exercise recommendations aimed at maintaining heart health should take into account biological differences between the sexes [51]. In addition, the level of physical activity among older adults at the start of the interventions is a variable to consider, since responses to the same program may differ between active older adults and those with insufficient physical activity [52]. However, the participants’ physical activity levels were not recorded in this study, limiting the generalizability of the results. Furthermore, the lack of a gold standard for determining the MQI suggests that these results should be interpreted with caution, as further research is needed.

### Equations for calculating the muscle quality index in older adults

Muscle quality includes microscopic and macroscopic aspects of muscle architecture and composition, underscoring its importance in assessing muscle strength and function in OA [53]. In this context, the scientific literature describes various methodologies for calculating the MQI [54] that use force measurements as a basis. These methodologies incorporate additional variables in the equations, such as gravitational force [8,55], BMI [56], or BM alone [57]. As previously mentioned, comparing MQI between women and men showed that men have a higher MQI (*p* < 0.0001), regardless of the equation used to calculate this indicator. Nevertheless, when correlating the functional tests and the GI of the GDLAM protocol with the two equations used to calculate the MQI, in both the dominant and non-dominant limbs, similar results are observed between the equations that include BMI and BM. Specifically, the literature has described that low muscle mass is associated with an increased risk of mortality, but also that BMI and ethnicity condition this relationship [58]. It has also been shown that the MQI, derived from strength and lean muscle mass, is a better indicator of muscle function than muscle strength alone [59]. Other studies have shown that both arm muscle area and arm fat area alone are better predictors of depressive symptoms in institutionalized OA compared to BMI [60]. These discrepancies, together with the results of the MQI calculated from the relationship between muscle strength and BMI or BM, require validation using a gold standard to support the precision and usefulness of these indices. Therefore, further exploration of these variables is considered necessary.

### Limitations

The main limitation to consider when interpreting the results is that participants were not stratified by their level of physical activity, a variable that could influence both muscle quality and the functional capacity assessed. Additionally, because participants who were physically unable to perform the tests were excluded, the present results characterize SPP completion time among older adults capable of performing the task, rather than the broader ability to rise from the floor, which would also include complete incapacity to do so. Furthermore, because the study is cross-sectional, it is not possible to establish causal relationships among the variables analyzed. Finally, the possibility of selection bias associated with the participant recruitment process should be considered. Moreover, in the regression models, the standardized residuals departed from normality and showed evidence of heteroscedasticity, a pattern commonly observed in positively skewed, time-based performance outcomes such as SPP; although these violations do not bias the point estimates of the regression coefficients, they may reduce the precision of the standard errors and confidence intervals, and future studies could consider robust or bootstrap-based estimation approaches to address this limitation.

## Conclusions

The results indicate that the MQI is associated with the time to stand up from the floor (SPP) among community-dwelling older adults. Males also outperformed females on the SPP test. The different MQI models were significantly associated with the time to stand up from the floor (SPP) in older adults capable of performing the task, even after adjusting for age, sex, stature, and fat mass percentage. In particular, models that used BM to calculate the MQI showed greater explanatory power compared to those based on BMI. Likewise, age showed a significant association with performance on the SPP test across all models, whereas sex showed a smaller contribution that varied by model. Taken together, these findings support an association between MQI and SPP performance, suggesting that the SPP test may serve as a practical, field-based indicator related to functional autonomy in older adults. However, this study did not evaluate the diagnostic accuracy or agreement of the SPP test against a reference measure of functional autonomy; therefore, these results should be interpreted as evidence of association rather than as validation of SPP as an alternative or substitute measure.

### Perspective

Future studies specifically designed to assess the diagnostic properties of the SPP test (e.g., sensitivity, specificity, or agreement with an established reference standard of functional autonomy) are needed to determine its potential role as a screening or assessment tool. Additionally, future research should design and implement personalized physical exercise programs that consider the MQI of men and women, focusing on maintaining and increasing MQI, given its positive association with FA in community-dwelling older adults.

### Supporting information

The database used in this study can be downloaded from the following link: https://doi.org/10.6084/m9.figshare.32470002
